# The Prevalence of Atherosclerosis in Those with Inflammatory Connective Tissue Disease by Race, Age, and Traditional Risk Factors

**DOI:** 10.1038/srep20303

**Published:** 2016-02-04

**Authors:** Francis J. Alenghat

**Affiliations:** 1 Section of Cardiology, Department of Medicine, University of Chicago, 5841 S. Maryland Ave., Chicago, IL, 60637, USA

## Abstract

Systemic inflammation promotes cardiovascular disease. Inflammatory connective tissue diseases (CTD) like lupus and rheumatoid arthritis associate with cardiovascular risk, but it is unknown whether particular groups of patients have enhanced propensity for atherosclerotic cardiovascular disease (ASCVD) associated with their CTD. Analysis of aggregate health record data at a large U.S. academic center identified CTD and ASCVD status for 287,467 African American and white adults. ASCVD prevalence in those with CTD was 29.7% for African Americans and 14.7% for white patients with prevalence ratios, compared to those without CTD, of 3.1 and 1.8, respectively. When different types of CTD were analyzed individually (rheumatoid arthritis; lupus; scleroderma; Sjögren Syndrome; dermatomyositis/polymyositis; unspecified/mixed CTD; other inflammatory arthropathy), increased ASCVD rates were found in nearly all subsets, always with higher prevalence ratios in African Americans. The prevalence ratio of ASCVD was particularly high in young African Americans. Furthermore, individuals lacking traditional cardiovascular risk factors had more ASCVD if they had CTD (prevalence ratio 2.9). Multivariate analysis confirmed a positive interaction between CTD and African-American race and a negative interaction between CTD and age. The factors driving the observed disproportionate CTD-associated ASCVD in African Americans, young adults, and those without traditional risk factors warrant further study.

Inflammation is a major contributor to atherosclerosis^1^. The intrinsic inflammatory component of atherosclerosis is supported by numerous analyses of cardiovascular primary and secondary prevention studies in which subjects with the highest inflammatory markers, such as C-reactive protein, carried cardiovascular risk rivaling those with the least favorable lipid profiles^2^,^3^,^4^,^5^. In recent years, clinical trials specifically targeting inflammation in cardiovascular disease have emerged, either completed with encouraging results (JUPITER for rosuvastatin in primary prevention and LoDoCo for colchicine in secondary prevention) or ongoing (CIRT for methotrexate in secondary prevention and CANTOS for canakinumab in secondary prevention)^2^,^3^,^6^,^7^. These trials are motivated by our understanding of the *local* inflammatory processes within the developing atheroma, but there is also substantial evidence that atherosclerosis is influenced by *systemic* inflammation. Patients with inflammatory CTD have greater systemic inflammation than what is found in the general population. For instance, compared to the highest quintile cutoff for C-reactive protein in a primary prevention population (~4 mg/l), the average is over three-fold higher (~14 mg/l) in patients newly diagnosed with rheumatoid arthritis (RA)^4^,^8^. Indeed, consistent with the role of inflammation in atherosclerosis, increased rates of cardiovascular disease (CVD) have been observed in populations with inflammatory CTD–most often described in RA and systemic lupus erythematosus^9^,^10^,^11^,^12^,^13^.

Still, there are several important questions about the connections between systemic inflammatory disease and atherosclerosis in need of clarification in order to impact evaluation and management of patients presenting with CTD. First, it is not established if different age groups or racial groups have different incremental risk conferred by CTD. Secondly, there remains uncertainty about the importance of traditional risk factors in the risk associated with CTD^13^,^14^. Finally, many of the prior studies have focused on populations with one specific CTD (often RA) and a broad collection of CVD that includes not only ASCVD but also heart failure and arrhythmia^9^,^11^, which are important clinical outcomes associated with certain CTDs^15^,^16^,^17^ but which may not necessarily respond to systemic inflammation in the same way as atherosclerosis. It is worth probing further the connection specifically between atherosclerosis and a set of connective tissue diseases that share common threads of systemic inflammation in order to identify particular groups of patients that have disproportionately greater ASCVD associated with their CTD.

Specifically regarding race, while it seems that, anecdotally, African American CTD patients frequently develop ASCVD, there is a dearth of formal understanding on any race-CTD interaction in ASCVD prevalence. The prior studies describing cardiac disease in patients with RA, for instance, were on largely white populations^10^,^18^. Furthermore, in the general population, the higher prevalence of ASCVD in African Americans is not completely understood^19^,^20^. While traditional risk factors are important, high rates of ASCVD in African American CTD patients, if present, would support the possibility that differences in inflammation play a role, as also suggested by slightly higher propensity for inflammatory cytokine production in the African American population at large^21^.

A cross-sectional analysis of a large, diverse patient population would help develop finer atherosclerotic disease risk estimations in those with and without CTD. Here, a systematically queried warehouse of de-identified data was used to examine the prevalence of inflammatory CTD and ASCVD at the University of Chicago, an urban medical center with large white and African American patient populations. Through analysis of routinely collected aggregate medical information, this evaluation sought to uncover age- and race-specific relationships between CTD and ASCVD, and to determine if CTD could also impact those who lack traditional cardiovascular risk factors.

## Results

The University of Chicago’s data warehouse of de-identified information includes clinical and demographic characteristics of over 300,000 adult patients. Of these, 287,467 patients comprised the analytic sample (Supplementary Figure S1): 158,355 who were identified as African American and 129,132 who were identified as white. Because patients identified as other races were a small fraction of the patient population, aggregate analysis based on these other races was not attempted.

**Figure 1:**
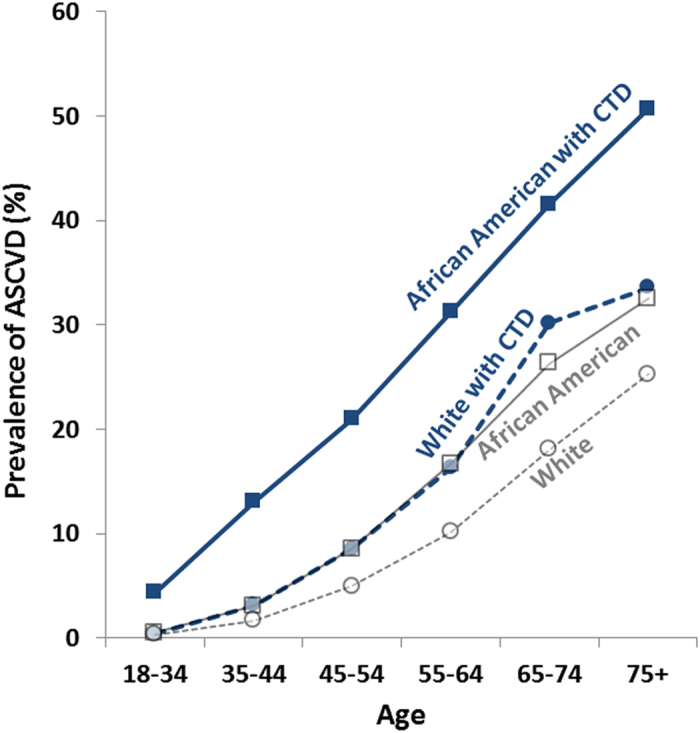
The prevalence of ASCVD in the general population and in those with CTD. ASCVD prevalence (gray) increases with age in the patient population at an urban academic center, with higher prevalence among African American compared to white patients. These prevalence rates, as well as the difference between white and African American patients, are consistent with other large datasets in this country. In the subpopulation with CTD (blue), the prevalence is higher than in the general population across all age groups. White patients with CTD have similar ASCVD prevalence as the general African American population, whereas the prevalence in African Americans with CTD is substantially higher. See Table 1 for details of age and race breakdown.

ASCVD, defined as including myocardial infarction, ischemic heart disease, angina, coronary artery disease, or atherosclerosis of any artery, was documented in 26,750 patients in this study. Of these patients, 15,899 were African American (prevalence 10.0%) and 10,851 were white (prevalence 8.4%). As expected, the prevalence of ASCVD increased with age, and the African American population had higher prevalence across all adult age groups (Fig. 1, gray). The observed prevalence of ASCVD, as well as the difference between white and African American patients, was consistent with other large contemporary statistics in the United States^19^,^22^,^23^. CTD, defined as including diagnoses of RA, systemic lupus erythematosus (SLE), systemic sclerosis, Sjögren syndrome, dermatomyositis, polymyositis, other inflammatory polyarthropathy, and unspecified diffuse or mixed connective tissue disease (UCTD/MCTD), was present in 8,747 patients (Supplemental Figure S1). In the CTD population, the prevalence of ASCVD was higher than in the general population across all age groups and among both races studied (Fig. 1, blue). African American adults with CTD had a prevalence of ASCVD of 29.7%, 3.1-fold higher than the prevalence in non-CTD African American adults (Table 1). White patients with CTD had a 14.7% prevalence of ASCVD, 1.8-fold higher than non-CTD white adults (Table 1). Half of the African American patients over age 75 with CTD also had ASCVD (50.7% prevalence, Fig. 1).

Among the different types of CTD, RA was the most common diagnosis, and many patients had more than one CTD diagnosis (Table 2). When the various forms of CTD contained within the main analysis were analyzed separately, the ASCVD rates were higher in all forms of CTD in African Americans and in all forms of CTD except isolated Sjögren Syndrome and isolated UCTD/MCTD in white patients (Table 2). In all forms of CTD, the ASCVD prevalence was higher in African American patients than in white patients, and the prevalence ratio (prevalence of ASCVD in the specific CTD group compared to ASCVD prevalence in the non-CTD group of the same race) was significantly higher in African Americans for nearly every CTD analyzed (Table 2). In both races, patients with more than one type of CTD had higher ASCVD prevalence (Table 2).

**Figure 2:**
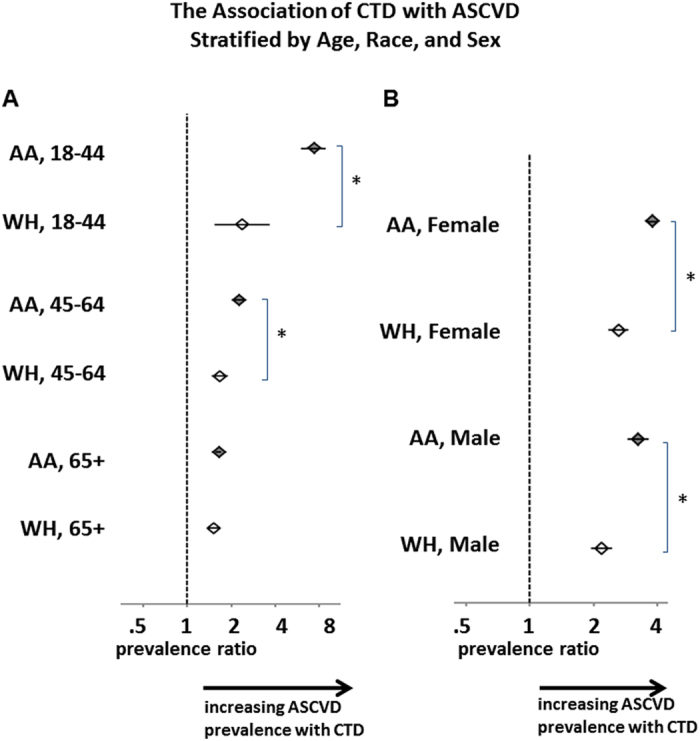
Prevalence ratios of ASCVD among age, race, and sex subgroups. For each indicated population, the ratio of ASCVD prevalence in those with CTD to those without CTD is shown. All populations shown have significant increases in ASCVD prevalence with CTD compared to without. (**A**) African American (AA) young and middle-aged adults with CTD have disproportionately increased prevalence of ASCVD, as compared to white subjects (WH) of the same age with CTD. (**B**) The disproportionate effect of CTD on African Americans applies to both sexes. Bars represent the 95% CI. *p < 0.001.

There was a 1.6-2.2-fold higher ASCVD prevalence in middle-aged and older adults with CTD compared to those without CTD (Table 1). Young adults with CTD (ages 18-44) had even more pronounced prevalence of ASCVD relative to their non-CTD peers. This effect in young adults compounded the effect of race: with a prevalence of 9.1%, African American young adults with CTD carried an ASCVD diagnosis 7.3 times more frequently than young African Americans without CTD (prevalence 1.2%) and 4.7 times more frequently than young white subjects with CTD (prevalence 1.9%) (Fig. 2A). The racial differences in the association between ASCVD and CTD was observed in young and middle aged adults (Fig. 2A), and they were present regardless of sex (Fig. 2B). Multivariate analysis with logistic regression models adjusted for age, sex, and race, along with interactions amongst these factors and with CTD, confirmed a positive interaction coefficient between African American race and CTD, as well as a negative interaction coefficient between older age groups and CTD, to demonstrate that the CTD-ASCVD association is stronger in African Americans and in the young (Supplementary Tables S1 and S2). There is no significant interaction between CTD and sex when adjusting for all collected demographic factors (Model 4, Supplementary Table S2).

**Figure 3:**
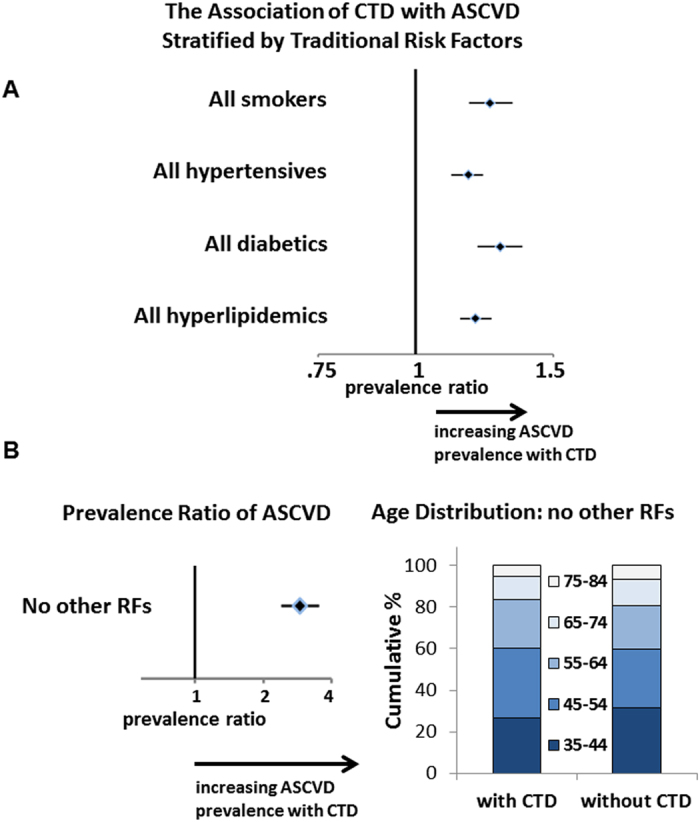
Prevalence ratios of ASCVD among subgroups by traditional ASCVD risk factors. (**A**) When accounting for any single traditional risk factor (RF), there remains ~1.2-1.3-fold more ASCVD in adults with CTD compared to those without. (**B**) In those over age 35 without hypertension, smoking, hyperlipidemia, or diabetes, there is ~3-fold more ASCVD observed in adults with CTD (4.5% prevalence of ASCVD) compared to those without (1.5% prevalence). There was no difference in the age distribution of these patients with vs. without CTD. Bars represent the 95% CI. All populations shown have significant increases in ASCVD prevalence in those with CTD compared to those without. See Table 1 for details and p values.

To probe the role of traditional cardiovascular risk factors in the association between CTD and ASCVD, subpopulations possessing a diagnosis corresponding to a traditional risk factor were analyzed. The prevalence of diabetes, smoking, hypertension, and hyperlipidemia was higher in the CTD population. With the exception of hyperlipidemia, the African American CTD population had higher prevalence of these risk factors compared to the white CTD population (African American CTD vs. white CTD: diabetes 20.2% vs. 6.5%; smoking 20.7% vs. 9.2%; hypertension 49.8% vs. 31.0%; hyperlipidemia 25.0% vs. 36.2%). A stratified approach showed that when controlling for a single risk factor at time, there remained elevated prevalence of ASCVD in those with CTD compared to those without (Fig. 3A). For instance, diabetics with CTD had a 10% absolute increase in ASCVD prevalence compared to non-CTD diabetics (43.2% vs. 33.7%; Table 1). Moreover, when examining patients over age 35 without any documented traditional risk factors, the overall prevalence of ASCVD was low, but those with CTD still had a 3-fold higher prevalence of ASCVD than those without (Fig. 3B). Importantly, this increased ASCVD rate associated with CTD in the absence of traditional risk factors was not due to a difference in age between the CTD and non-CTD patients (Fig. 3B).

## Discussion

The traditional cardiovascular risk factors of hypertension, diabetes, hyperlipidemia, and smoking are well established^24^,^25^,^26^,^27^. In recent years there has been increasing recognition that inflammatory states like CTD can confer additional risk, but there remains considerable uncertainty regarding whether the risk associated with CTD is conferred by an enhancement of traditional risk factors or if CTD itself represents true residual risk^13^,^14^,^28^,^29^. Furthermore, much of the prior work on this topic has focused on RA, although smaller investigations of lupus and other CTD have been done^10^,^11^,^30^. In addition to traditional cardiovascular risk factors, prolonged steroid therapy, chronicity and activity of disease, and postmenopausal status appear to raise risk specifically in CTD patients^31^,^32^. Many pathogenic mechanisms for increased atherosclerosis are possible in patients with CTD and likely overlap with the pathophysiology of the specific CTD itself; these include systemic inflammatory responses, autoantibody and immune complex mediated processes, and increased inflammatory cytokines from activated T cells both systemically and locally at the atheroma^33^. Some of these mechanisms, along with secondary increases in sympathetic tone, may also account for other clinical important cardiovascular outcomes in CTD patients, including heart failure and arrhythmia^15^,^16^,^17^,^34^.

The approach taken here was to assess a heterogeneous group of CTDs that have systemic inflammation as a common thread even though the molecular and cellular pathogenesis of each disease is distinct. Furthermore, in contrast to past work which has focused on a broad assessment of cardiovascular disease, the current analysis focused solely on cardiovascular disease attributable to atherosclerosis. The data support the association between ASCVD and CTD, and they lend credence to regarding CTD as a risk factor for ASCVD. To date, a limited number of cardiovascular risk scores, including QRISK2 and the recent ERS-RA, incorporates history of RA, while another (ATACC-RA) is in development^32^,^35^. No other CTD besides RA is currently incorporated into any widely accepted cardiac risk calculators. In the current analysis, higher rates of ASCVD occur across a range of diverse types of CTD, not only RA and SLE, but also Scleroderma, Dermatomyositis/Polymyositis, other inflammatory arthropathies, as well as Sjögren Syndrome and UCTD/MCTD in African Americans. The advantage of understanding all of these CTDs as potential risk factors for atherosclerosis would be to have a lower threshold for initiation of primary prevention and for initiation of inflammatory disease modifying therapy.

The analysis has uncovered a difference in the strength of association of CTD with ASCVD between two major racial groups in the United States. Observational studies of RA and CVD have been done, but without significant numbers of African American patients, and an interaction with race has not been previously described^10^. The extent to which the difference between races observed here reflects social or healthcare differences is unknown, but disparities—potentially avoidable differences in health among groups of differing social advantage^36^—are well documented between races for both CVD and CTD^20^,^37^,^38^. Indeed the uneven effect of CTD on African Americans unfortunately serves to exacerbate the underlying ASCVD difference observed in the African American population at large. The higher prevalence of traditional cardiovascular risk factors in African American CTD patients may contribute to the ASCVD difference observed, although this study cannot prove this point, and the role of pathophysiological differences versus social/healthcare differences in driving the development of these risk factors is uncertain^39^,^40^,^41^. Socioeconomic status could be involved in the observed differences as well^42^, and future work on verifiable individual-level records (as opposed to the aggregate data here) could clarify this further. The largest genetic study of RA to date did not include African Americans or others of African descent, and further interactions between genetic factors and cardiovascular risk in this CTD population cannot be surmised at this time^43^. Intriguingly, small studies in South Africa have not uncovered an association between RA and atherosclerotic burden in African black patients (who historically have lower ASCVD than the African white population, the opposite of the U.S.)^44^,^45^. QRISK2 was derived in the UK without African Americans—compared to white residents, subjects of Black African and Black Caribbean descent in the UK have lower CVD risk^35^. Taken together, these observations would imply there are modifiable factors that could be addressed to reduce the disproportionate cardiovascular effects of CTD on African American patients.

Without other comprehensive studies in the United States on a wide spectrum of CTD and the demographic subpopulations most prone to ASCVD, generalizability would be speculative, but the current findings are probably most applicable to metropolitan areas in the United States. As for the smaller rural population in the United States, given that rural and urban ASCVD outcomes are not widely different^46^, the present results could be applicable in those settings as well. In contrast, the experience in South Africa suggests that the same relationship between white and African-descent populations is not generalizable globally.

The disproportionate effect of CTD on younger age groups can be potentially explained by the relative lack of other traditional risk factors at those ages. As the population ages, the accrual of hypertension, diabetes, hyperlipidemia, and years of smoking partially overshadow the effect or preexisting CTD, but in the young, CTD is at the fore. Young African Americans have a particularly notable association between CTD and ASCVD, and the explanation is not known. It is possible that the types of CTD in these patients are particularly aggressive, less adequately treated, less amenable to treatment, or associated more strongly with other traditional risk factors^47^,^48^,^49^.

The non-conventional approach used here was chosen in order to analyze the routinely collected clinical information over a large population to uncover connections between a heterogeneous set of systemic inflammatory diseases and atherosclerosis. As demonstrated, in the current era of electronic health records, systems designed for meaningful use of extant information can allow for identification of previously unidentified relationships and for generation of hypotheses for further investigation. This work should serve as a starting point for more endeavors in this field through in-depth analysis of the relative contributions from individual types of CTD and the reason for the disproportionate effect of CTD on the atherosclerotic burden of African Americans.

There are several important limitations to the current investigation. First, it relies on diagnostic codes found in the electronic record. In this type of aggregate analysis, diagnoses cannot be confirmed with further chart review, but there is no reason to expect that such inconsistencies would bias the findings in one direction over another, particular with the large number of cases assessed. It is quite possible that the true association between ASCVD and CTD in our patient population is stronger than what was observed due to under-recognition of cardiovascular disease in CTD patients^18^. Secondly, race classifications are reliant on a specifically coded race field in the EMR. Race is not confirmed with further chart review or text analysis, but, again, there is no reason to expect that such inconsistencies would bias the findings in one direction over another. Both these limitations could be addressed in future work by curating individual identified records at this and other medical centers with large white and African American populations. Next, the focus was on ASCVD prevalence and not on incident ASCVD once the CTD diagnosis was established. While the approach prevents formal assessment of cardiovascular risk, it may better reflect pathophysiology of chronic disease. Because the mechanisms underlying both ASCVD and CTD are chronic and smoldering with subclinical disease that can exist for prolonged periods, it is not necessarily the case that CTD-associated ASCVD events should appear only after establishment of the CTD diagnosis. Still, to address ASCVD incidence, there is ongoing work to identify aggregate matched cohorts and follow them through the EHR over 5 years. Fourth, while multivariate analysis of CTD and demographic data was performed and confirmed a CTD-race and CTD-age interaction, the aggregate data were not well-suited for further parsing of the impact of traditional risk factors due to overstratification. Multivariate analysis on curated identified records should be able to delineate better the contribution of traditional risk factors versus inherent CTD-mediated risk. Finally, the correlations between ASCVD and CTD, as with any correlation, do not define the pathophysiologic connection or causal relationship between these disease states, which will likely be clarified by basic science work as well as the effects of highly-targeted CTD therapies in clinical trials.

Thus, in future work, it will be worth exploring further the role of therapy in CTD-associated ASCVD^50^. Higher degrees of inflammation correlate with atherosclerotic progression in CTD patients^14^,^21^,^51^. In some studies, access to disease-modifying antirheumatic drugs is associated with less cardiovascular events in patients with RA^52^,^53^,^54^. As for traditional cardiovascular medications, statin therapy in RA patients does seem to reduce cardiovascular events^55^. Analyzing aggregate data for treatment effects in a broad cross-sectional analysis such as this was prevented because accounts of timing and dosing of anti-inflammatory therapy were not possible. Still, these prior studies along with the current data, especially in young patients, point toward potential benefit of early risk factor identification and control of inflammation as important goals in the management of connective tissue disease in order to prevent atherosclerosis. From a clinical perspective, it should be considered appropriate and standard to fully assess patients newly diagnosed with CTD for all the traditional cardiovascular risk factors and employ risk calculation with one or more calculators, with upward adjustment for the presence of CTD, in order to have a well-informed discussion with the patient regarding initiation of both pharmacologic (statin, aspirin, blood pressure control) and non-pharmacologic (smoking cessation, exercise, weight loss, heart-healthy diet) primary prevention measures. For example, given that that AHA/ACC guidelines suggest statins for patients with calculated 10-year risk of >7.5%^56^, and since African Americans ages 35-44 with CTD have an 8.2% rate of ASCVD (albeit prevalence, not incidence, from Table 1), African Americans with CTD could reasonably consider moderate-intensity statin therapy at age 35.

Taken together, the current findings show that CTD is associated with higher ASCVD prevalence in white and African American patients in all adult age groups, in both sexes, in those with at least one documented traditional cardiovascular risk factor, and in those with none. This association is accentuated in African Americans, young adults, and those without traditional risk factors. The data presented here challenge the boundaries of our understanding of systemic inflammation, cardiovascular risk, and how co-existing disease can associate with atherosclerosis disparately between different groups of patients. These insights could particularly inform how to evaluate African American or young patients who present with CTD.

## Methods

Data were obtained using the i2b2 platform (Boston, Massachusetts), applied to the Clinical Research Data Warehouse (CRDW) at the University of Chicago. This platform allows for de-identified access to the electronic medical record system to glean aggregate data on patients based on diagnoses primarily using International Classification of Diseases, Version 9 (ICD9) diagnosis codes and demographics. With this tool, applied to all records from January 2008 through April 2014, sets of patients at University of Chicago Medicine were defined with diagnoses corresponding to an array of connective tissue diseases encompassed by ICD9 710 (diffuse diseases of connective tissue) and ICD9 714 (RA and other inflammatory polyarthropathies). These two codes include systemic lupus erythematosus, Sjögren syndrome, dermatomyositis, polymyositis, RA, unspecified inflammatory polyarthropathy, and unspecified or mixed diffuse connective tissue disease (UCTD/MCTD). Other diagnoses included in these codes but without sufficient sample sizes to analyze individually are Felty’s syndrome, eosinophilia myalgia syndrome, juvenile chronic polyarthritis, and chronic postrheumatic arthritis. Likewise, sets of patients in the CRDW were defined with diagnoses corresponding to an array of atherosclerotic states, encompassed by ICD9 410 (acute myocardial infarction), ICD9 411 (ischemic heart disease), ICD9 412 (old myocardial infarction), ICD9 413 (angina pectoris), ICD9 414 (other forms of chronic ischemic heart disease), and ICD9 440 (atherosclerosis). These diagnoses include coronary, aortic, renal, peripheral, and other arterial atherosclerosis, but do not explicitly include stroke or cerebrovascular disease, as those ICD9 codes do not differentiate well between atherosclerotic, embolic, and hemorrhagic cerebrovascular disease. Finally, traditional cardiovascular risk factors were assessed using ICD9 272.0, 272.1, 272.3, 272.4 (hypercholesterolemia); ICD9 250 (diabetes mellitus); ICD9 401 (essential hypertension); and ICD9 V15.82, ICD9 305.1, HCPCS G8690, HCPCS G8686, HCPCS G8455, CPT 1034F (current or past tobacco/smoker).

Age, race, and sex were determined by the electronic medical record’s race, age, and sex fields. Demographic factors collected included race (Black/African American or White; other races in the population were not well enough represented to facilitate aggregate analysis), age group, and sex. Ethnicity, coded as a separate variable without reliability, was not collected. Those identifying as Hispanic and White would be classified as White, and those identifying as Hispanic and Black would be classified as Black/African American. If a multiracial patient was coded as either White or Black (regardless of the true nature of the race) that is how the patient would have been recorded. If the multiracial patient was coded as Other, then he/she would not be included in the analysis. There were no other inclusion or exclusion criteria.

Prevalence ratios on aggregate data were calculated with 95% confidence intervals (CIs) as previously described, and p-values were calculated using the χ^2^ test^57^. The p values for intergroup comparisons of prevalence ratios were calculated using the χ^2^ test on total cases in each group paired with excess cases associated with CTD. Multivariate analysis was performed with logistic regression models adjusted for race (Model 1), age (Model 2), sex (Model 3), or all three combined (Model 4). Interactions between these demographic variables and with CTD were included in the models. Interaction coefficients, confidence intervals and p values from these models were reported. These analyses were conducted using Stata 13.

## Additional Information

**How to cite this article**: Alenghat, F. J. The Prevalence of Atherosclerosis in Those with Inflammatory Connective Tissue Disease by Race, Age, and Traditional Risk Factors. *Sci. Rep*. **6**, 20303; doi: 10.1038/srep20303 (2016).

## Supplementary Material

Supplementary Information

## Figures and Tables

**Table 1:** ASCVD Prevalence in Patients with and without CTD.

	Total	with CTD			without CTD			Prev. Ratio	95% CI	p^*^
	n	n	ASCVD	ASCVDRate (%)	n	ASCVD	ASCVD Rate (%)			
*Race*										
** African American**	158,335	4661	1383	29.7	153,674	14,516	9.4	**3.1**	[3.0–3.3]	<0.001
** White**	129,132	4086	600	14.7	125,046	10,251	8.2	**1.8**	[1.7–1.9]	<0.001
*Age*										
** 18–34**	84,520	1015	25	2.5	83,505	346	0.4	**5.9**	[4.0–8.9]	<0.001
** 35–44**	44,625	1224	99	8.1	43,401	1018	2.3	**3.4**	[2.8–4.2]	<0.001
** 45–54**	49,432	1814	265	14.6	47,618	3123	6.6	**2.2**	[2.0–2.5]	<0.001
** 55–64**	45,728	1830	427	23.3	43,898	5545	12.6	**1.8**	[1.7–2.0]	<0.001
** 65–74**	34,394	1512	550	36.4	32,882	6942	21.1	**1.7**	[1.6–1.8]	<0.001
** 75+**	28,768	1352	617	45.6	27,416	7793	28.4	**1.6**	[1.5–1.7]	<0.001
*Traditional Risk Factors*											**										
** None (age 35+)**	181,256	2362	107	4.5	178,894	2792	1.6	**2.9**	[2.4–3.5]	<0.001											
** Diabetes**	24,247	1368	591	43.2	22,879	7713	33.7	**1.3**	[1.2–1.4]	<0.001											
** Smoking**	26,235	1515	599	39.5	24,720	7862	31.8	**1.2**	[1.2–1.3]	<0.001											
** Hypertension**	54,603	3588	1190	33.2	51,015	14,499	28.4	**1.2**	[1.1–1.2]	<0.001											
** Hyperlipidemia**	44,496	2643	1098	41.5	41,853	14,581	34.8	**1.2**	[1.1–1.2]	<0.001											

*p values are derived from χ^2^ tests for comparisons between CTD and non-CTD populations. Intergroup comparisons of the CTD-associated ASCVD prevalence ratios shown here indicate significantly disproportionate effect of CTD on African Americans, young adults, and those without traditional risk factors (p <0.001 by χ^2^ test).

**Table 2:** ASCVD Rates by Type of CTD.

CTD type	African American				White				p**: AA vs WH
	n	ASCVD rate (%)	Prev. Ratio*	95% CI	n	ASCVD rate (%)	Prev. Ratio*	95% CI	
**Rheumatoid Arthritis (RA)**	**2409**	**37.4**	**4.0**	**[3.7–4.2]**	**1841**	**20.5**	**2.5**	**[2.3–2.7]**	**<0.001**
RA and no other CTD	1579	39.0	4.1	[3.9**–**4.4]	1185	20.9	2.6	[2.3**–**2.9]	<0.001
RA plus other CTD	830	34.2	3.6	[3.3**–**4.0]	656	19.8	2.4	[2.1**–**2.8]	<0.001
**Systemic Lupus Erythematosus (SLE)**	**1706**	**26.0**	**2.7**	**[2.5–3.0]**	**1037**	**15.5**	**1.9**	**[1.6–2.2]**	**<0.001**
SLE and no other CTD	955	21.9	2.3	[2.1**–**2.6]	542	12.4	1.5	[1.2**–**1.9]	<0.001
SLE plus other CTD	751	31.2	3.3	[3.0**–**3.7]	495	19.0	2.3	[1.9**–**2.8]	<0.001
**Scleroderma**	**391**	**35.5**	**3.8**	**[3.3–4.3]**	**451**	**21.1**	**2.6**	**[2.1–3.1]**	**<0.001**
Scleroderma and no other CTD	140	25.7	2.7	[2.1-3.6]	234	17.9	2.2	[1.7**–**2.9]	0.097
Scleroderma plus other CTD	251	41.0	4.3	[3.7**–**5.0]	217	24.4	3.0	[2.4**–**3.8]	<0.001
**Sjögren Syndrome**	**424**	**27.6**	**2.9**	**[2.5–3.4]**	**699**	**12.6**	**1.5**	**[1.3-1.9]**	**<0.001**
Sjögren Syndrome and no other CTD	127	15.7	1.7	[1.1**–**2.5]	295	4.1	0.5	[0.3**–**0.9]	<0.001
Sjögren Syndrome plus other CTD	297	32.7	3.5	[2.9**–**4.1]	404	18.8	2.3	[1.9**–**2.8]	<0.001
**Dermatomyositis/Polymyositis (D/P)**	**340**	**32.6**	**3.5**	**[3.0–4.0]**	**265**	**17.7**	**2.2**	**[1.7–2.8]**	**<0.001**
D/P and no other CTD	163	30.7	3.2	[2.6**–**4.1]	158	14.6	1.8	[1.2**–**2.6]	<0.001
D/P plus other CTD	177	34.5	3.6	[3.0**–**4.5]	107	22.4	2.7	[1.9**–**3.9]	0.044
**UCTD/MCTD** (and no other CTD)	**202**	**21.3**	**2.3**	**[1.7–2.9]**	**457**	**7.9**	**1.0**	**[0.7–1.3]**	**<0.001**
**Other inflammatory arthropathy** (and no other CTD)	**248**	**35.5**	**3.8**	**[3.2–4.4]**	**308**	**12.7**	**1.5**	**[1.2–2.1]**	**<0.001**

*Prevalence ratio is the ratio of ASCVD rate in the specific CTD type to that in the non-CTD population of the same race. In African American patients, every major CTD type is associated with a significantly higher ASCVD rate compared to the non-CTD population. In white patients, every CTD type except isolated Sjögren Syndrome and isolated UCTD/MCTD is associated with significantly higher ASCVD rate compared to the non-CTD population. **p values are derived from χ^2^ tests for comparisons between African American (AA) and white (WH) ASCVD rates for each CTD. The rates and prevalence ratios of ASCVD are significantly higher in African Americans compared to white patients in nearly all analyzed CTD types.
