# Supplementary material for: The Prevalence of Atherosclerosis in Those with Inflammatory Connective Tissue Disease by Race, Age, and Traditional Risk Factors
Source: Sci Rep. 2016 Feb 4;6:20303. doi: 10.1038/srep20303 (PMC4740809; doi:10.1038/srep20303)
Supplement: Supplementary Information [file srep20303-s1.pdf]

# Supplementary Materials

## The Prevalence of Atherosclerosis in Those with Inflammatory Connective Tissue Disease by Race, Age, and Traditional Risk Factors

Francis J. Alenghat, MD, PhD

**Affiliation:**

Section of Cardiology, Department of Medicine, University of Chicago, 5841 S. Maryland Ave., Chicago, IL, 60637, USA

**Address for Correspondence:**

Francis J. Alenghat, MD, PhD, Section of Cardiology, Department of Medicine, University of Chicago, 5841 S. Maryland Ave., Chicago, IL, 60637, USA

Tel: (773) 834-0705

Fax: (773) 702-8875

Email: alenghat@uchicago.edu

**Figure S1: Schematic representation of the studied patient population.**

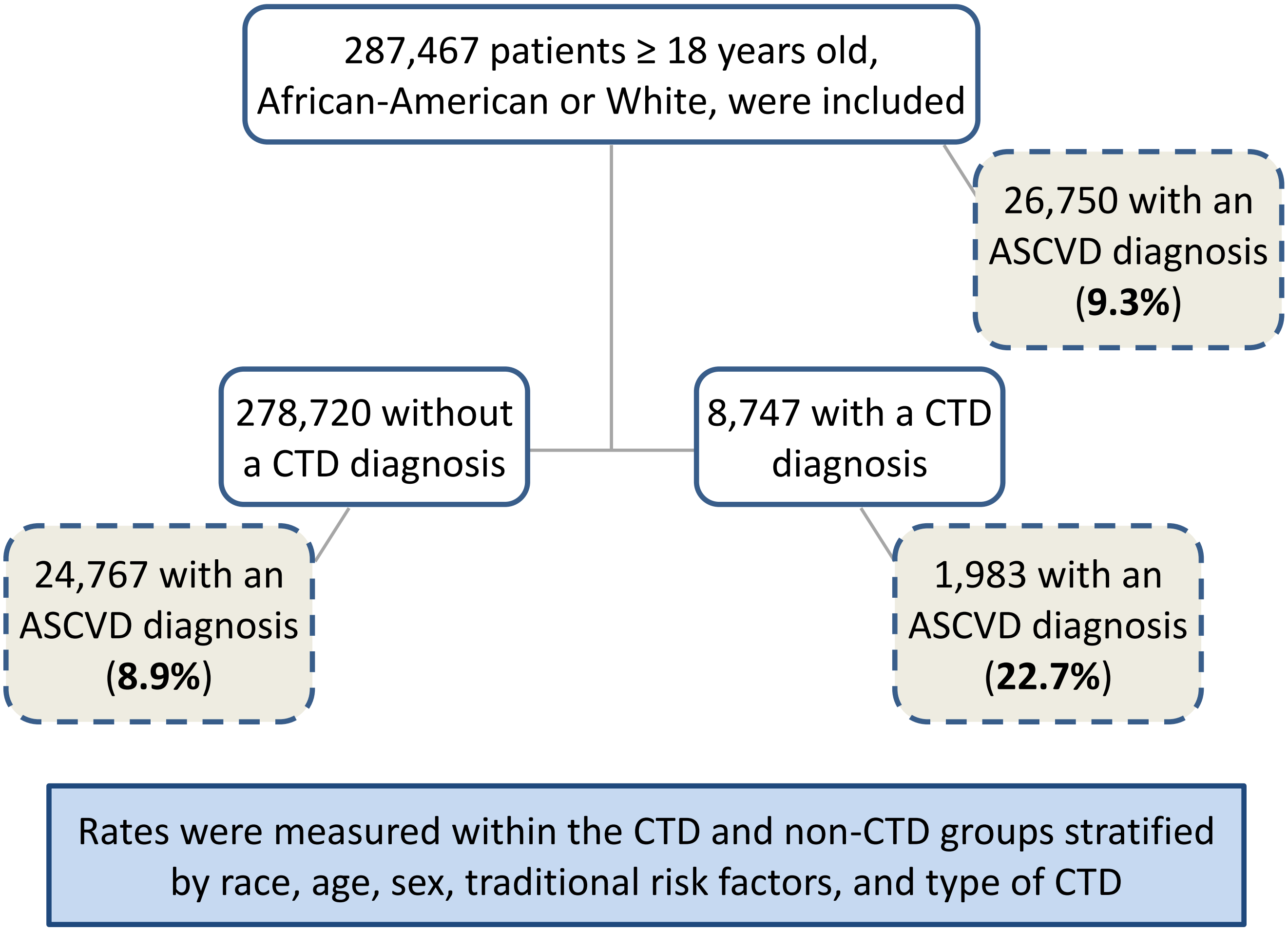

**Table S1. Demographic Interactions with the CTD-ASCVD Association, Models 1-3**

| Interaction Pair | Model | Interaction Coefficient | 95% CI | p |
|---|---|---|---|---|
| CTD*White | 1 | 0 | | |
| CTD*AA | 1 | 0.71 | [0.59 to 0.83] | < 0.001 |
| | | | | |
| CTD*[age 18-44] | 2 | 0 | | |
| CTD*[age 45-64] | 2 | -1.06 | [-1.27 to -0.84] | < 0.001 |
| CTD*[age 65+] | 2 | -1.09 | [-1.30 to -0.88] | < 0.001 |
| | | | | |
| CTD*Female | 3 | 0 | | |
| CTD*Male | 3 | -0.16 | [-0.29 to -0.02] | 0.024 |

Coefficients of interaction between CTD and demographic groups in their association with ASCVD derived from logistic regression models adjusted for race (Model 1), age (Model 2), and sex (Model 3). There is a significant positive interaction between CTD and AA race and a significant negative interaction between CTD and the older age groups.

**Table S2. Demographic Interactions with the CTD-ASCVD Association, Model 4**

| Interaction Pair | Interaction Coefficient | 95% CI | p |
|---|---|---|---|
| CTD*White | 0 | | |
| CTD*AA | 0.18 | [0.04 to 0.31] | 0.009 |
| | | | |
| CTD*[age 18-44] | 0 | | |
| CTD*[age 45-64] | -1.09 | [-1.30 to -0.87] | < 0.001 |
| CTD*[age 65+] | -1.28 | [-1.49 to -1.06] | < 0.001 |
| | | | |
| CTD*Female | 0 | | |
| CTD*Male | -0.04 | [-0.19 to 0.10] | 0.557 |

Coefficients of interaction between CTD and demographic groups in their association with ASCVD derived from multivariate analysis using a logistic regression model adjusted for race, age, and sex (Model 4). There is a significant positive interaction between CTD and AA race and a significant negative interaction between CTD and the older age groups.
